# Membrane structures and functional correlates in the bi-segmented eye lens of the cephalopod

**DOI:** 10.1242/bio.060445

**Published:** 2024-08-29

**Authors:** Justyn W. Regini, Naoto Yagi, Robert D. Young, Hidetoshi Tanioka, Shigeru Kinoshita, Masato Hoshino, Kentaro Uesugi, Keith M. Meek, Andy T. Augousti, Carlo Knupp, Barbara K. Pierscionek, Andrew J. Quantock, Gerald F. Elliott

**Affiliations:** ^1^ Structural Biophysics Group, Cardiff Centre for Vision Science, School of Optometry and Vision Sciences, Cardiff University, Maindy Road, Cardiff, CF24 4HQ, Wales, UK; ^2^ SPring-8, Japan Synchrotron Radiation Research Institute, 1-1-1, Kouto, Sayo-cho, Sayo-gun, Hyogo, 679-5198 Japan; ^3^ Department of Ophthalmology, Kyoto Prefectural University of Medicine, Hirokoji Kawaramachi, Kyoto, 602-0841, Japan; ^4^ Faculty of Science, Engineering and Computing, Kingston University London, Friars Avenue, London SW15, UK; ^5^ Faculty of Health Medicine and Social Care, Medical Technology Research Centre, Anglia Ruskin University, Bishop Hall Lane, Chelmsford CM1 1SQ, UK

**Keywords:** Eye lens, Cell membrane complexes, Cephalopod

## Abstract

The cephalopod eye lens is unique because it has evolved as a compound structure with two physiologically distinct segments. However, the detailed ultrastructure of this lens and precise optical role of each segment are far from clear. To help elucidate structure–function relationships in the cephalopod lens, we conducted multiple structural investigations on squid. Synchrotron x-ray scattering and transmission electron microscopy disclose that an extensive network of structural features that resemble cell membrane complexes form a substantial component of both anterior and posterior lens segments. Optically, the segments are distinct, however, and Talbot interferometry indicates that the posterior segment possesses a noticeably higher refractive index gradient. We propose that the hitherto unrecognised network of membrane structures in the cephalopod lens has evolved to act as an essential conduit for the internal passage of ions and other metabolic agents through what is otherwise a highly dense structure owing to a very high protein concentration.

## Teaser

**Summary:** The cephalopod eye lens is unique because it has evolved as a compound structure with two physiologically distinct segments. To help elucidate structure-function relationships in the cephalopod lens we conducted multiple structural investigations on squid.

## INTRODUCTION

Throughout evolutionary history, it has been proposed that the eye has evolved independently over forty times, and this highly specialised organ represents an excellent example of parallel evolution (Luitfried and Mayer, 1977; Land, 1984; Gehring, 2005). The classic anatomical and morphological research that suggested multiple evolutionary origins of the animal eye has been challenged by more recent work in genetics that indicates that evolution of the eye may have a monophyletic origin (Treisman, 2004; Gehring, 2012; Gehring, 2014). In nearly all species, the main function of the eye lens is to focus light onto the retina and, as such, its optical performance is largely dictated by the visual requirements of the particular animal, which in turn are determined by environmental factors such as habitat, feeding and reproduction. Interestingly, although they have developed separately, the camera-type eyes of mammals and cephalopods display remarkable similarities. Their lenses are composed of fibre-like cells synthesised in concentric layers, like rings in a tree, with the progressive growth of new cells over existing tissue, which continues throughout life creating a chronology of cell fibre layers of different ages throughout the tissue. Also, within their lenses, both mammals and cephalopods have used the same mechanism of utilising crystallin proteins from pre-existing enzymes or stress-related chaperone proteins. In both cases, the crystallin proteins, which are contained within the lens fibre cells and which show the highest concentration towards the centre of the lens, are responsible for the maintenance of short-range, liquid-like order in the cytoplasm, helping to achieve the refraction of light and lens transparency in the visible spectrum (Delaye and Tardieu, 1983).

An understanding of the evolution of the material and optical properties of the lens and its crystallins is complex because mammalian lenses contain varying combinations of three different crystallin sub-types, α−, β- and γ-. This is compounded by the fact that each of the mammalian crystallin proteins has numerous isoforms (Harding, 1991). A more homogeneous tissue in terms of protein diversity is the squid lens, which possesses a single major type of crystallin – S-crystallin – as 70% of its soluble protein, alongside very low amounts of Ω-crystallin (Zinovieva et al., 1993; Sweeney et al., 2007a). The S-crystallins of cephalopods are descended from, but not identical to, a single-copy glutathione S-transferase (GST) liver enzyme (Tomarev et al., 1995). However, the S-crystallins from squid lens do not demonstrate any glutathione S-transferase activity (Zinovieva et al., 1993). Rather, their main role in the lens is a structural one, in maintaining short-range structural order. The S-crystallins have a mean of about 81% amino acid homology to each other and 40% amino acid homology to a liver expressed GST enzyme, and thus their overall quaternary structure appears to be conserved (Tomarev et al., 1995).

From a functional perspective, the main optical difference between the eyes of species residing in water and those that live in air is the negligible refractive (i.e. focusing) power of the cornea in water. The cornea of the eye is the most powerful refractive element in air. However, in aquatic environments the cornea loses the vast majority of its refractive power due to the similarity of the refractive indices of the cornea, and of water. Thus, the eye lens in water has to become the main refracting element of the eye supplying almost all of the eye’s focusing power. As a result, the eyes of animals with such lenses are much more spherical in shape in comparison to land-based or flying animals (Sivak and Warburg, 1983). If such a spherical lens with a high optical power were to be made from a material with a uniform refractive index, optically it would result in a high degree of spherical aberration and blurred vison. Most aquatic animals have evolved to have spherical lenses, which pose a graded refractive index profile to overcome this problem (Pierscionek and Regini, 2012). By having a higher protein concentration in the centre of the lens that decreases radially, a radial gradient of lens protein concentration is achieved. Thus, in such a lens, a lower refractive index occurs at the peripheral margins of the lens, and increases towards the centre where it is at its maximum. In the outer layers of the cortex of human lenses, the protein concentration is approximately 200 mg/ml, and 450 mg/ml in the central nucleus (Harding, 1991). On the other hand, the lenses of aquatic species have much higher protein concentration – 900 mg/ml in teleost fish (Mirarefi et al., 2010) – and a sharper refractive index gradient. In a lens with a uniform refractive index, light is refracted at the lens surface, the light is not focused any further until the point at which the ray leaves the lens. In a lens with a gradient index, as light passes through the lens, it is continually focused.

The quality of the image that is produced at the retina, and the contribution the refractive index gradient makes to the overall refractive power of the lens, depends on the type of gradient which differs with species (Pierscionek and Regini, 2012). The gradient itself is largely dependent on the lens crystallins, their concentrations and contribution to the lens’ refractive index. In many aquatic species such as cephalopod, the steep refractive index gradient in the lenses allows for a small eye size, with high sensitivity and excellent imaging. Indeed, some species of squid are thought to possess a visual acuity comparable to that of humans (Sweeney et al., 2007b).

Teleost fish lenses are approximately spherical. Their high crystallin concentration results in a lack of pliability and hence different mechanisms of altering focussing power in response to visual demand (i.e. accommodation) are required. For terrestrial animals, lenses shaped as highly oblate spheroids, the process of accommodation is achieved by changing the shape and curvature of the lens via the ciliary muscles. For non-pliable fish lenses, on the other hand, accommodation takes place by the axial movement of the lens via the retractor lentis muscle, thus changing the distance to the retina (Sivak, 1985). The squid lens is strikingly different from both, however, in that it is a compound lens with two main structural elements: an anterior plano-convex segment and a larger posterior segment with a greater diameter (Fig. S1). The two are joined by a septum, which has an associated ciliary body (Willekens et al., 1984; West et al., 1995). It is thought that each structural element of the cephalopod lens develops from separate ectodermal sources (Packard, 2008), unlike in most vertebrate lenses, which develop from one source. Also, it is significant that the anterior and posterior segments are electrically uncoupled so that there is no physiological communication or flow of ions between them (Jacob and Duncan, 1981).

When considering lens transparency, it is important to note that, although the lens grows throughout life, most of its cells have lost their organelles, and their nuclei are pyknotic and located away from the path of light to avoid scattering. Lens cells – also known as lens fibres owing to their shape and lack of organelles – rely on anaerobic glycolysis. The very low metabolic activity and lack of a blood supply means that they exist with significant metabolic constraints. Mature lens fibres no longer exhibit metabolic activity and do not possess ion channels or Na^+^/K^+^ pumps to create a negative membrane potential needed to maintain their steady state volume (Mathias et al., 1997). This problem is particularly acute in aquatic lenses with very high protein concentrations where there is very little free water, and in these conditions, passive diffusion alone is not able to support ionic and metabolic homeostasis of central fibre cells (Mathias et al., 1997). Electrical impedance measurements taken at the lens surface, however, indicate that there is an ionic current mainly of Na^+^ ions (Robinson and Patterson, 1982), and it has been suggested that this flow represents the external part of a circulating ionic current that drives an internal fluid circulatory system from the equator of the lens (which is furthest from the optical axis) to the poles (which the optical axis crosses), thus allowing the lens fibres to maintain homeostasis and to remain transparent. This model has not gained universal acceptance in the field, however, largely because circulating fluid flows have been difficult to measure directly inside the lens (Beebe and Truscott, 2010; Donaldson et al., 2010).

At present, the reasons why cephalopods have evolved with a compound lens and the precise optical role of each segment are far from clear. The mechanism of accommodation is also not fully understood. Unlike teleost fish, cephalopods do not have a retractor lentis muscle to move the lens in an anterior-posterior direction with respect to the retina. However, a study using infrared photoretinoscopy to measure the accommodation of live cuttlefish demonstrated that the animals focused selectively only in the frontal visual field, with no change in refraction in the lateral field of view, leading to a model of accommodation in which the lens moves axially with respect to the retina (Schaeffel et al., 1999). The current study uses synchrotron X-ray scattering and interferometry, alongside transmission electron microscopy (TEM), to investigate the ultrastructure and optical characteristics of the cephalopod lens and allude to the conditions which likely allow the ionic and metabolic homeostasis in the centre of the tissue.

## RESULTS

### Small-angle synchrotron X-ray scattering (SAXS)

**Fig. 1.**
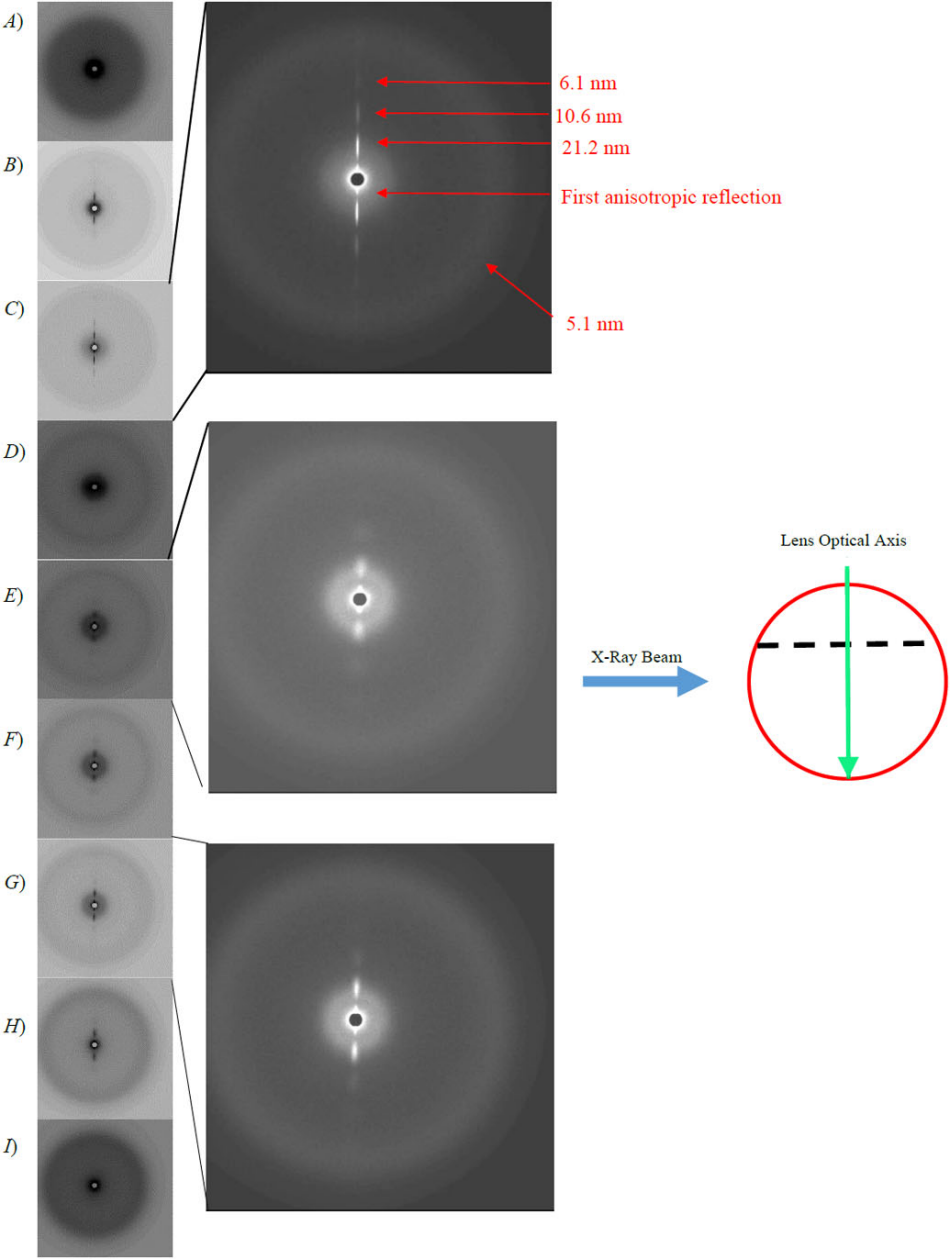
**A series of X-ray diffraction patterns summarising the one-dimensional scan A of a whole squid lens.** Each individual pattern represents 1 mm along the length of the scan. Panels A to C are from the anterior segment, D is from the septum and E-I are from the posterior segment. Expanded images of Figures 1 (C), (E) and (G) with inverted grey scales are also shown for clarity. The schematic diagram shows a side view of a whole squid lens (red circle) the dotted line represents the septum, in relation to the optical axis of the lens (green area) and the direction of the X-ray beam (blue arrow) in this scan.

**Fig. 2.**
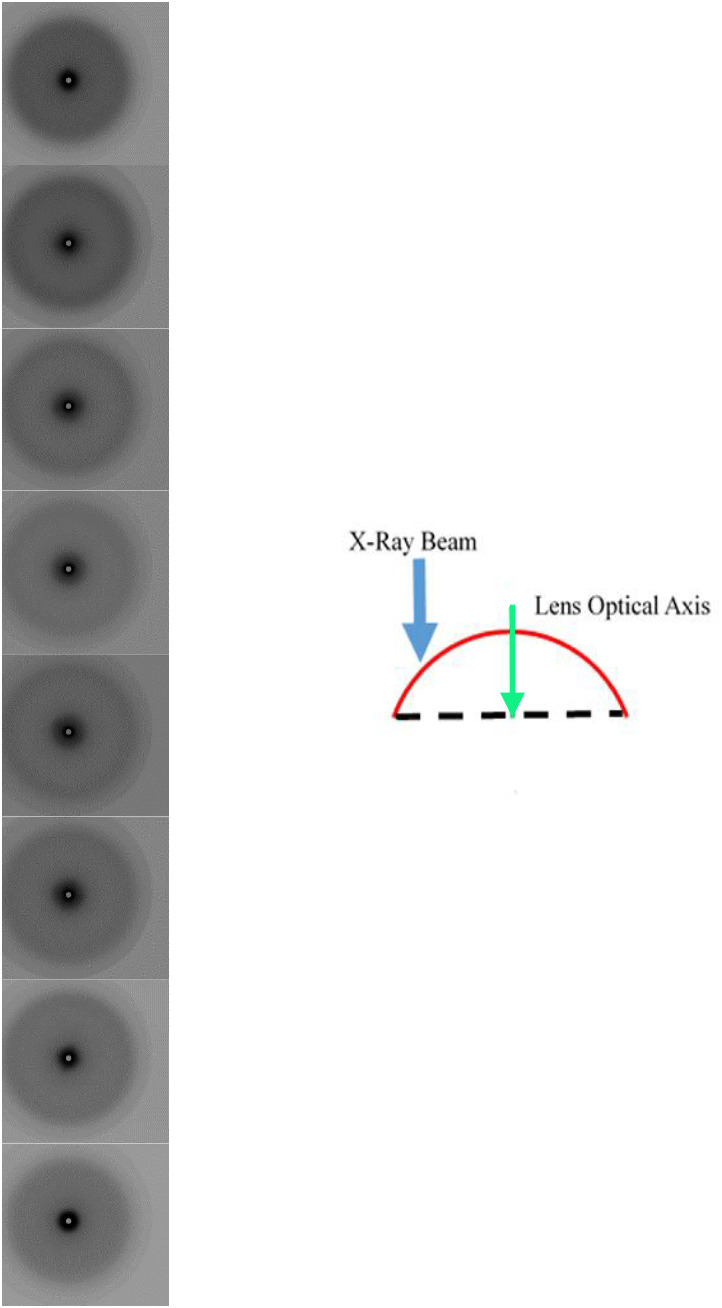
**A series of X-ray diffraction patterns summarising the one-dimensional scan B of anterior segment with the X-ray beam parallel to the optical axis of the lens.** Each individual pattern represents 1 mm along the length of the scan. The schematic diagram shows a side view of the anterior segment of the lens (red curve and dotted line), in relation to the optical axis of the lens (green area) and the direction of the X-ray beam (blue arrow) in this scan.

**Fig. 3.**
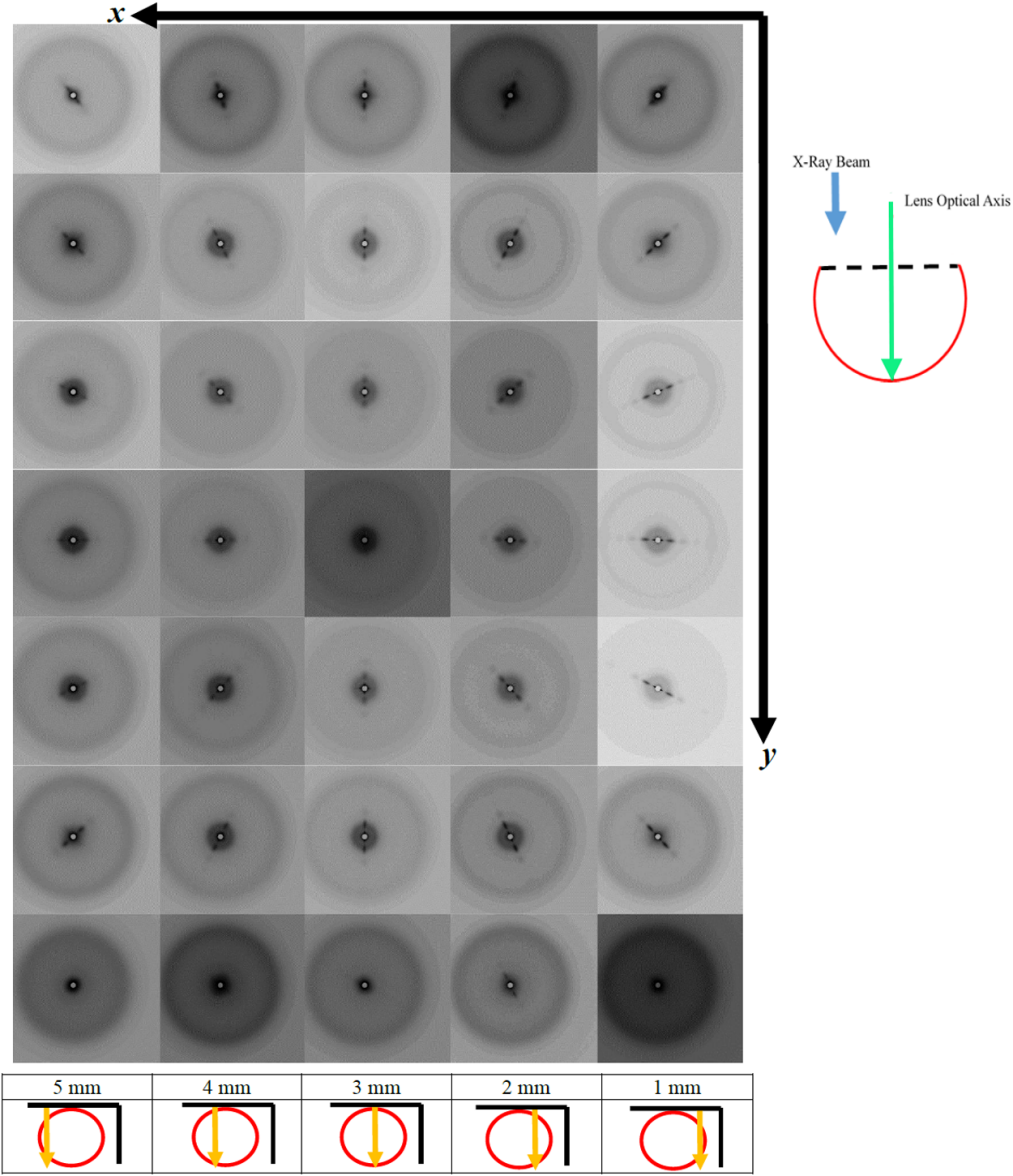
**A series of X-ray diffraction patterns summarising the two-dimensional scan C of posterior segment with the X-ray beam parallel to the optical axis of the lens.** Every 1 mm along the *x*-axis of scan C, a series of images is shown which correspond to a distance of 1 mm in the y-direction of scan C. The numbers 1-5 correspond to mm from the edge of the lens. The top right hand schematic diagram shows a side view of the posterior segment of the lens (red curve and dotted line), in relation to the optical axis of the lens (green area) and the direction of the X-ray beam (blue arrow) in this scan. The schematic diagram at the bottom of the column of each scan shows a top-down view of the posterior segment of the lens (red circle) and the direction of the scan (orange arrow) in relation to the *X* and *Y* axis (black lines) of the two-dimensional scan.

Two isolated squid lenses from the same animal (either whole or divided into anterior and posterior sections) were raster scanned by a 50µm-diameter X-ray beam in 100μm steps. Two of the scans, A and B (Figs 1 and 2), were one-dimensional, while scan C (Fig. 3) was two-dimensional. In scan A, one whole lens was traversed along its meridian, so that the beam was at right angles (orthogonal) to the optical axis of the lens and passed though both the anterior and posterior segments. For the second lens, the anterior and posterior segments were separated and investigated individually. In scan B, the lens’ anterior section was examined via a one-dimensional scan, with the X-ray beam oriented in a direction parallel to the optical axis of the lens. In scan C, the posterior segment was investigated using a two-dimensional raster, again with the beam co-axial with the lens’ optical axis. This scan provided 80×80 SAXS patterns in the *x* and *y* directions and a total of 6400 data points.

Fig. 1 shows an abridged version of scan A, with 1 mm steps between each displayed SAXS pattern. This traverses the whole of the lens with the beam orthogonal to the optical axis, passing through the anterior and posterior segments of the lens. In this figure, each image is taken at a distance of 1 mm along the equatorial plane. The full scan comprising all the SAXS patterns separated by 100 µm increments can be seen in a video in the online supplementary materials (Movie S1)*.* In Fig. 1, panels A-C are from the anterior segment, D from the septum and E-I from the posterior segment. It should be noted that, in nearly all the scans, the SAXS patterns recorded at the periphery of the lens (i.e. Fig. 1A and I) show only an isotropic diffraction ring. As the beam passes toward the centre of the lens away from periphery, the SAXS patterns similarly contain isotropic scattering, but also intense anisotropic scatter in the vertical direction (Fig. 1B,C,E,F,G and H). Fig. 1D is the exception, with this image taken at the location of the septum between the two segments. It only displays diffuse isotropic scattering. For clarity, expanded images of Fig. 1C,E and G are shown with inverted grey scales. From the expansion of Fig. 1C, it can be seen that the anisotropic reflections are long and narrow and most intense on either side of the circular beam stop. In this image, the innermost of these reflections was difficult to measure due to its proximity to the circular beam stop at the centre of all the SAXS patterns. The second of these reflections away from the beam stop corresponds to a spacing of 21.2 nm and then appears to repeat at 10.6 nm and 6.1 nm in the third and fourth reflections. The fourth reflection has a relatively low intensity and was only observed in the anterior segment. The isotropic outer ring is seen, in this case, at 5.0 nm, and corresponds to the interference function observed in SAXS patterns from mammalian lenses. As with the anterior segment, the posterior segment from scan A also displays reflections that are both isotropic and anisotropic. The expanded Fig. 1E and G show the images taken at, respectively, distances of 1 and 3 mm away from the septum. Again, the most intense anisotropic reflections are observed next to the beam stop and they continue in a series further out. However, these anisotropic reflections are less well defined than those from the anterior segment. In Fig. 1E, the second anisotropic reflection is fairly broad and round. In conjunction with this reflection, another isotropic interference reflection is observed, which corresponds to the same spacing as the second anisotropic reflection. In the SAXS patterns obtained at distances further away from the septum (expanded Fig. 1G), the second anisotropic reflection becomes slightly more elongated in appearance.

Fig. 2 shows an abridged version of the one-dimensional scan B taken across an isolated anterior segment of a squid lens, with the X-ray beam oriented parallel with the optical axis. Again, each image in this figure is taken at intervals of 1 mm*.* The full scan comprising all the SAXS patterns separated by 100 µm increments can be seen in a video in the online supplementary materials (Movie S2). Here, the dominant feature of the SAXS patterns is the first interference function, which most likely corresponds to the packing of the S-crystallin proteins within the lens fibre cells. Similar X-ray reflections are observed in SAXS patterns obtained from mammalian lenses and have been attributed to the packing of α−, β- and γ-crystallins within the fibre cells. Notably, however, the anisotropic reflections, which were seen when the X-ray beam was passed through the anterior lens in a direction orthogonal to the optical axis (Fig. 1), did not appear in any of the scan B images when the beam passed through the anterior segment in a direction parallel to the lens’ optical axis. This suggests that the structure or structures that give rise to the anisotropic reflections are unidirectional and in a plane at right angles to the optical axis of the anterior segment. The most obvious feature of scan B is the change in the size of the first interference function from the periphery to the centre. This corresponds to a spacing of 5.0 nm at each edge and 4.9 nm in the centre. Fig. S2 shows the spacing of the interference function taken from the summary images of scan B as a function of position within the scan. These values fall and rise quite steeply at distances of 1 to 3 mm, and from 5 to 8 mm, respectively.

A summary of the two-dimensional scan C of the isolated posterior segment with the X-ray beam parallel to the optical axis of the lens is shown in Fig. 3. SAXS patterns are presented at 1 mm intervals in both the *x* and *y* directions. The full scan comprising all the SAXS patterns separated by 100 µm increments can be seen in a video in the online supplementary materials (Movie S3). All the X-ray patterns in this scan display similar features to those taken from the posterior segment in scan A (Fig. 1E-H), with comparable, broad anisotropic reflections. The most striking feature of Fig. 3, however, is that the anisotropic reflections rotate around the centre of the pattern on either side of the meridian of the posterior segment of the lens. The one-dimensional scan at the 3 mm position on the *x* axis is taken along the meridian of the lens in the *y*-direction of the two-dimensional scan. Here, in nearly all the images, the anisotropic reflections are vertical within the X-ray patterns. The exceptions to this are patterns taken at the peripheries and, more interestingly, at the very centre of the posterior segment, where only the interference function is observed. The anisotropic reflections rotate in a clockwise direction as *y* increases in scans to the right of the 40th scan position, whereas they rotate in an anti-clockwise direction in scans to the left of the 40th. Figs S3 and S4 show the spacings of the second and third anisotropic reflections, along with the interference functions measured from the summary images of the 40th scan plotted as a function of distance along the scan. The spacings of the second and third anisotropic reflections show very little in the way of overall trend across the posterior segment and vary from 24.2 to 26.7 nm, and from 10.4 to 11.7 nm, respectively. These values are slightly larger than those measured from the corresponding anisotropic reflections from the anterior segment of the lens. The spacings from the interference function (Fig. S4), range from 5.2 nm at the periphery to 4.7 nm in the centre. When compared to the values from the anterior segment (Fig. S2), it can be seen that the posterior segment measurements are closer together across most of the lens and have a shallower profile toward the periphery.

### X-ray Talbot interferometry

**Fig. 4.**
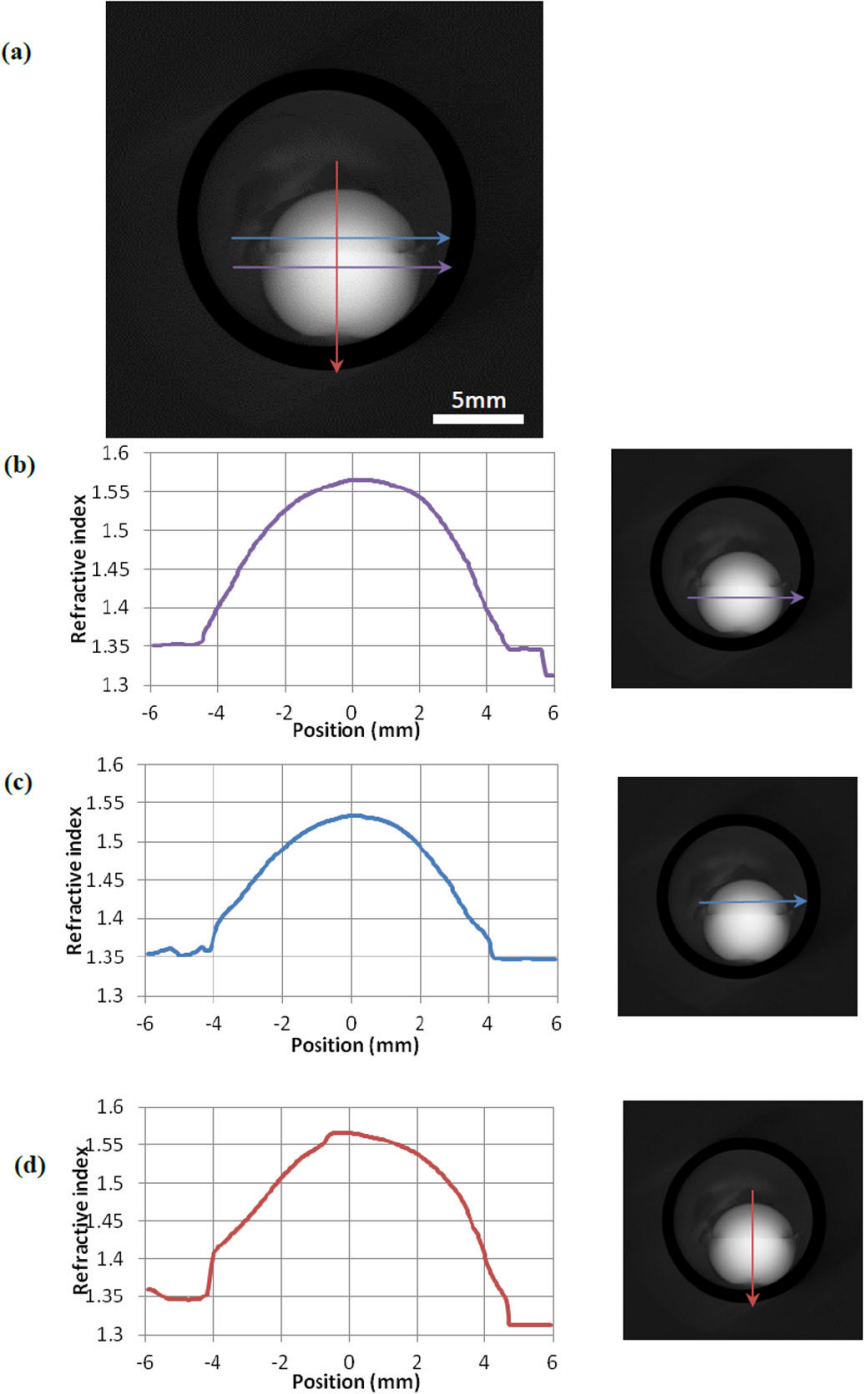
**X-Ray Talbot Interferometry of a whole squid lens.** (A) The image of a whole squid lens showing the anterior segment and posterior segments. The position of the equatorial plane is marked with blue and purple arrows in the anterior and posterior segments respectively. The optical axis, along which the sagittal refractive index profiles were measured, is indicated with a red arrow. (B) The refractive index profile in the equatorial plane of the posterior segment and the image of a whole squid lens showing the location of the profile. (C) The refractive index profile in the equatorial plane of the anterior segment and the image of a whole squid lens showing the location of the profile. (D) The refractive index profile along the optical axis (sagittal plane) of both the anterior and posterior segments and the image of a whole squid lens showing the location of the profile.

X-ray Talbot interferometry, which we have previously used to directly measure the refractive index gradients of the intact lens (Hoshino et al., 2021), was performed on whole isolated lenses held in agarose gels to maintain hydration. Measurements were obtained in two planes: one along the optical axis of the lens (the sagittal plane) and the other along the plane orthogonal to this (the equatorial plane). Fig. 4A shows the image of a whole squid lens in which the anterior and posterior segments are clearly defined. The arrows indicate the planes along which the refractive index profiles were measured. Fig 4B and C show the refractive index profiles measured in equatorial planes of the posterior and anterior segments, respectively. Although similar in that the refractive index profiles are broadly symmetrical in shape, the slope of the anterior segment is shallower than that of the posterior segment and has a lower peak value of 1.53 compared with 1.57. These profiles are remarkably smooth compared to those of other species that we have investigated. In our previous studies of porcine, ranine, murine, newt and piscine lenses, we found discontinuities and fluctuations in the refractive index profiles, which it was suggested may be linked to changes in growth rates (Hoshino et al., 2021). As can be seen from Fig. 4D, the refractive index profile along the optical axis (sagittal plane) in both segments of the squid lens is very different. It is characterised by being highly asymmetrical, with the profile in the anterior segment being almost linear. The profile in the anterior segment, on the other hand, resembles a more familiar shape which we observed in our previous studies (Hoshino et al., 2011). The abrupt increase in the profile at position −1 mm on the *x* axis in this figure indicates where the two segments meet at the septum. The asymmetry of this profile indicates that the anterior segment of the squid lens has a lower protein concentration than the posterior segment, and that the optical characteristics of the two segments are different.

### Transmission electron microscopy (TEM)

**Fig. 5.**
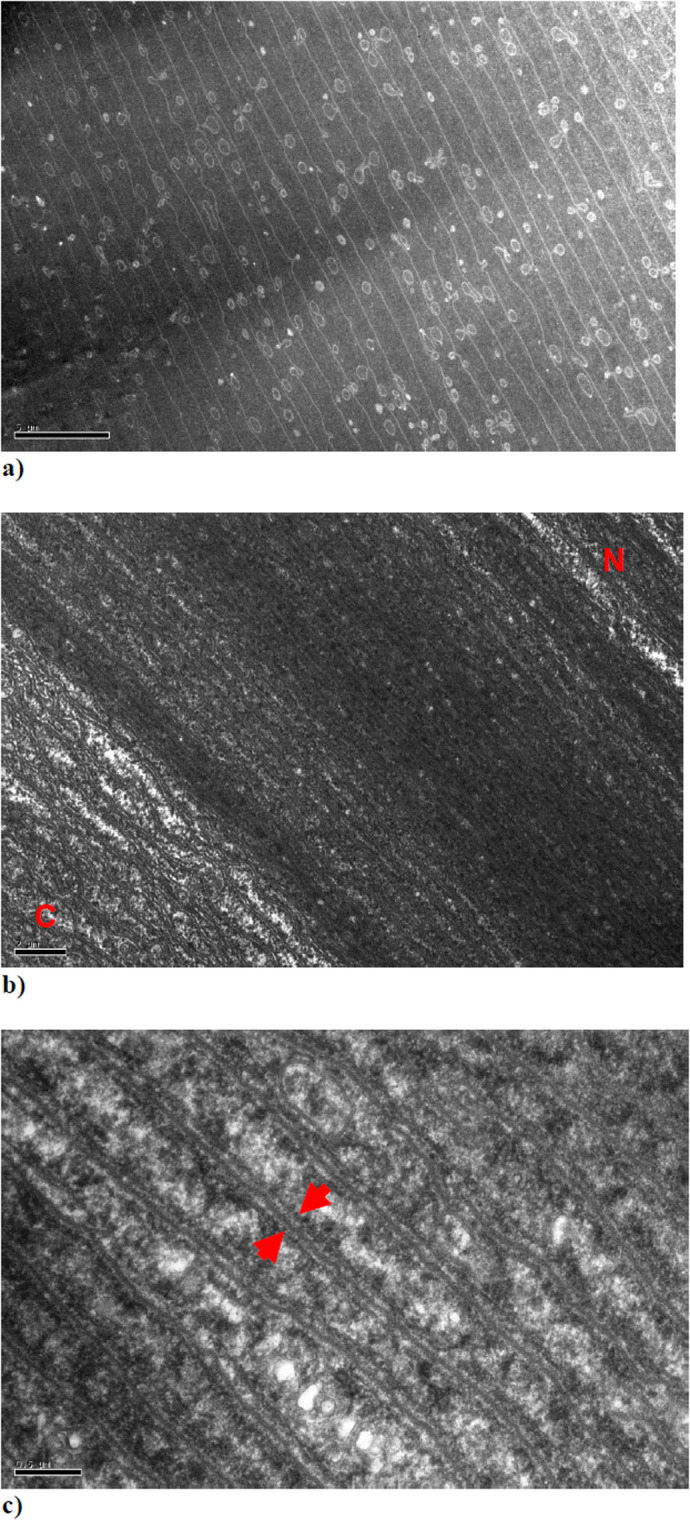
**TEM images from the anterior segment of a squid lens from a section cut parallel to the direction of the fibres.** The red letters C and N indicate the area of the image in which the newly accrued immature lamellae are located towards the outer cortex (C) and the more mature lamellae towards the nucleus (N) of the anterior segment. The red arrows in Fig. 5C indicate what appear to be an adjacent pair of membrane structures separated by a slightly darker channel.

TEM images of ultrathin sections of the anterior segment of a squid lens cut parallel to the main axis of the lens fibres indicate that they are approximately 1µm wide (Fig. 5). The circular and ovoid structures are the ball and socket connections of the interdigitating system that hold the fibres together. This image shows features that are very similar to those seen in TEM images from lenses of other species, where the sections have been cut in a plane parallel to the direction of the fibres (Vrensen et al., 1992; Taylor et al., 1996; Jongebloed and Kalicharan, 1998). No structures with a spacing or periodicity that could account for the strong isotropic reflections seen in the SAXS patterns are present. In Fig. 5B, individual sheets or lamellae run diagonally from the top left to bottom right of the image, with each lamella bounded on both sides by darkly banded structures that run parallel to each other. The bottom left corner of the image shows newly accrued immature lamellae (and hence fibre cells) which may be identified from being lighter in contrast (likely because of a lower protein concentration) and from being more invaginated in appearance than the more mature tissue. Therefore, in this case the centre of the anterior segment is in the direction of the top right corner of the image. A higher magnification image shown in Fig. 5C shows that there are three distinct structures in between the lamellae. On each surface of a lamella is a distinct thin band, which is slightly darker than the interior of the lamella. Between these adjacent structures is another thin band of darker contrast. Measurements for these darker bands from several high magnification images gave a mean spacing of 21.9 nm.

### Computer modelling

**Fig. 6.**
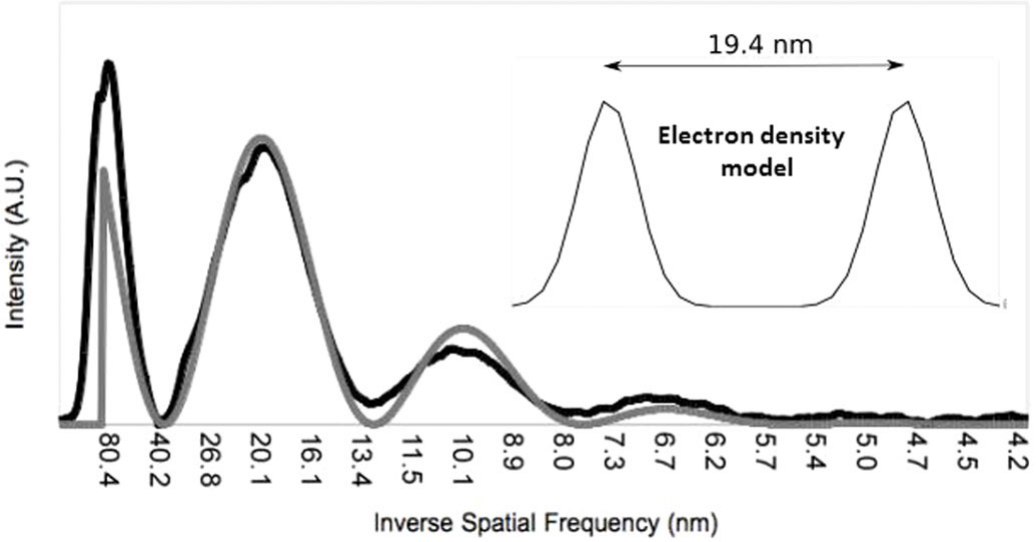
The background-subtracted X-ray intensity profile (black line) and calculated diffraction pattern (grey line) from the electron density model in the inset.

Using computer modelling techniques, we performed analysis of the experimental X-ray data from the very intense anisotropic reflections taken from the anterior section in scan A. Eleven individual X-ray patterns, similar to those of Fig. 1C, were averaged and the intensity profile along the length of the reflections taken. Fig. 6 shows the X-ray intensity profile (black line) along with the Fourier transform (grey line) generated using a one-dimensional electron density model (inset) which is suggested by the structures shown in Fig. 5C, plotted as a function of inverse spatial frequency. It was found that an approximately Gaussian curve gave a better fit to the data. The generated curve fits the experimental data almost exactly and the optimal distance between the two Gaussian curves was found to be 19.4 nm. From the intensity profile and the generated best fit, it is clear that they both follow a classic Cardinal Sine function. Therefore, the inner reflection in scan A (Fig. 1C) is the zeroth order reflection, the second reflection (21.2 nm) is the first order, the third reflection (10.6 nm) is the second order and the fourth reflection (6.1 nm) is the third order. The values of the distance across the membrane structures we have obtained from the three different techniques we have employed are remarkably similar: with 21.2 nm from X-ray diffraction, 21.9 nm from TEM and 19.4 nm from the computer simulation.

## DISCUSSION

Previous structural studies of the cephalopod lens have mainly centred on investigations of the surface structure using scanning electron microscopy (SEM). (Willekens et al., 1984; West et al., 1995), To our knowledge, this is the first study to investigate the internal structure with the use of both X-ray techniques and TEM. Previous low-angle X-ray diffraction studies have been shown to be able to probe the internal structures of intact, isolated mammalian lenses (Regini et al., 2004; Regini and Meek, 2009). These earlier studies show X-ray diffraction patterns with only one diffuse isotropic scattering reflection, with no other structural sampling. To demonstrate this, Fig. S5 shows an X-ray diffraction pattern from a bovine lens. The putative interpretation of this reflection is that it is an interference function and originates from the average nearest neighbour spacing between the crystallin proteins (Regini et al., 2004). In an intact mammalian lens, the reflection indexes to a spacing of about 15 nm, depending on the species, the age and hydration of the sample. In contrast, it is clear from the strongly diffracting anisotropic reflections observed in the current X-ray diffraction experiments (Figs 1 and 3) that some sort of structures with a high degree of ordering and periodicity exist in squid lens, but which either do not exist or are unable to be detected by this technique in the lenses of other species so far studied. From our TEM data, we observe that the banded structures between the lamellae of fibre cells (Fig 5B,C) have a striking resemblance to a cell membrane complex (CMC), with two dark bands that are possibly lipid layers on either side of a less electron dense channel. Moreover, the orientation and differing curvature of these structures in the different segments of the squid lens may help to elucidate the nature of the anisotropic reflections in scan A and C (Figs 1 and 3, respectively), and their absence in scan B (Fig. 2).

CMCs are known to be present in a number of tissues such as hair and skin, occurring as two separate lipid layers at the surfaces of adjoining cells. The central channel is thought to contain glycoproteins that act as a cement to hold the two separate bilayers together (Subczynski et al., 2017). It also acts a channel along which ions and nutrients may flow. In the human lens, the fibre cell membrane contains cholesterol bilayers within the plane of the cell membrane surrounded by liquid crystalline lipid membrane bilayers (Subczynski et al., 2017).

However, the high level of cholesterol in lens fibre cell membranes is not well understood and has not been measured in the squid lens, but given the increase in membrane cholesterol with progression from the cortex to the nucleus of the lens, found in some mammalian lenses, it has been suggested that this acts to maintain similar fluidity levels throughout the lens (Borchman et al., 1996). The membrane organisation of cholesterol may also be essential for transparency (Regini et al., 2004).

Strongly diffracting anisotropic equatorial X-ray reflections were first observed and attributed to CMCs from the myelin coating of nerve fibres by Worthington (Blaurock and Worthington, 1966; Worthington, 1969; Worthington and King, 1971). He postulated that it is the lack of electron density in the central channel of the CMC that gives rise to the very strong X-ray reflections. They have also been observed in X-ray patterns from hair fibres, which have a CMC in the cuticle and bear a striking resemblance to the reflections seen in this study (Kreplak et al., 2001; Ohta et al., 2005). The values of the distance across the membrane structures obtained from the three different techniques employed are remarkably similar: 21.2 nm from X-ray diffraction, 21.9 nm from TEM and 19.4 nm from the computer simulation. Of note, an early morphological study by Arnold (Arnold, 1966) reported structures 22 mµ (i.e. 22 nm) in diameter in the developing squid (*Loligo pealii*) lens, which were postulated to be microtubules, but which match the dimensions of CMCs that we find present throughout the adult squid lens. As noted earlier, at the edges of all the scans only the interference function is observed and not the anisotropic reflections. These are the regions where immature fibres are laid down, and it is likely that the membrane structures do not become fully developed until the lamellae have the full complement of proteins.

The subspherical curvature of lamellae of fibre cells, and hence the features between them, which may be CMCs can account for the left- and right-handed rotation of the anisotropic reflection on either side of the meridian in scan C of the posterior segment. The concentric nature of these structural membrane features and lamellae also explains both the vertical reflections observed along the meridian and the horizontal reflections along the equator of the two-dimensional scan (Fig. 3). Similar rotating low-angle X-ray reflections have been observed in two-dimensional arrays of fibrillar systems, which are centrosymmetric in nature (Yagi et al., 2006). The change in the spacing of the interference function along the length of scan C (Fig. S4) is consistent with there being a refractive index gradient with closer packing of the S-crystallins in the centre of the posterior segment than at the periphery. The reduction in the nearest neighbour crystallin separation observed in the squid lens is explained by the smaller size of S-crystallin when compared with the larger mammalian crystallins (Delaye and Tardieu, 1983), which allows closer packing of the protein molecules.

As scan A was taken along the meridian of the whole lens orthogonally to the optical axis, the vertical orientation of the anisotropic reflections is constant in both segments, consistent with structural features that resemble CMCs and lamellae possessing a plano-convex orientation in the anterior segment and sub-spherical in the posterior segment (Fig. 1). However, the reflections in the posterior segment are less elongated and more rounded than those of the anterior segment (Fig. 1C,E and G). This is due to the increased amount of curvature of the membrane structures in the posterior segment. In conjunction with this reflection, a second interference function is observed, which indexes on to the same spacing as the second anisotropic reflection, indicating that the membrane structures occur in many different directions throughout this segment but have a preferred orientation along the meridian. The plano-convex structure of the anterior segment explains the elongated and very intense nature of the equatorial anisotropic reflections, as the X-ray beam is able to sample the less electron-dense central channel of the membrane structures for relatively long distances between the lamellae. Moreover, the lack of any observed anisotropic reflections in the one-dimensional scan B, where the X-ray beam passed through an isolated anterior segment parallel to the optical axis, gives further support for the plano-convex orientation of the membrane structures.

Non-uniform concentric, lamellar biological structures, created by new synthesis over existing tissue and thereby forming a chronological growth record, are often found in nature, for example in the trunks of trees, in pearls, as well as in the eye lens. The presence of large numbers of membrane structures in very high protein concentration aquatic lenses would explain how these lenses have evolved to allow the essential transport of ions and nutrients from the periphery to the nucleus, thus being able to maintain transparency. But how does the evolution of the cephalopod lens impact on its optical function? The two main investigations have been those on cuttlefish accommodation mentioned earlier (Robinson and Patterson, 1982) and those on squid and octopus from the laboratory of Sivak (Sivak, 1982, 1991). In these studies, the authors explain and model the accommodation by axial movement of the lens. However, both groups are unable to account for the total amount of accommodation from axial movement alone. Our interferometry data (Fig. 4D) show that the anterior segment has a less steep refractive index gradient with a lower maximum index indicating lower concentrations of protein than in the posterior segment. This is backed up by the larger range in spacings of the interference function measured in the anterior segment, especially towards the periphery (Fig. S2). Importantly, this may cause the anterior segment to be more pliable than the posterior. It is known that cephalopods have a circular ciliary body which is connected close to the septum, so it is likely that the anterior segment can, to a limited extent, be deformed, which may occur in conjunction with movement in the anterior direction. Indeed, it may be that this segment could be deformed in a similar fashion to mammalian lenses, which would clearly be facilitated by the physiological and mechanical isolation of the two segments mentioned above.

## MATERIALS AND METHODS

### Specimens

Two adult squid (*Todarodes pacificus*) were purchased from Nishiki market, Kyoto, Japan and transported on ice to the SPring8 synchrotron, a journey of about 2 h, where they were stored overnight at 4°C. The next day, two lenses of one squid were examined by SAXS on beamline 40XU. One lens was studied intact, with the X-ray beam passed at right angles to the visual axis through anterior and posterior sections of the lens in a raster fashion (Fig. 1). The other lens was separated into its anterior and posterior sections, each of which was studied with the X-rays passed through the tissue in a direction parallel to the optical axis (Figs 2 and 3). The lenses from the other squid were photographed (Fig. S1) and prepared for TEM as described below. Typically, squid at Nishiki market are sold within 48 h of being caught, thus the SAXS data were collected roughly 72 h post-mortem. The main body of the squid was about 40 cm long. For the interferometry experiments, a live squid was purchased from a restaurant in Himeji, near SPring8, with data collected within 30 mins of death. Unless otherwise stated, experiments were conducted at room temperature.

### Small-angle X-ray scattering (SAXS)

SAXS experiments were conducted on the low-angle, high flux beamline, 40XU, at the SPring8 synchrotron radiation facility, Hyogo Prefecture, Japan. Prior to the experiments, the optical clarity of the whole isolated lens and the lens that had been separated into its two sections, anterior and posterior, was confirmed by placing the specimens on graph paper and ensuring that the pattern of the grid lines could be clearly discerned. For data collection, samples were wrapped in clingfilm, secured in a specimen holder and positioned in the path of a 50 µm diameter X-ray beam. A 3-metre-long camera was used, with exposure times of 0.1 s each. Calibration was achieved using the 67 nm repeat from wet rat tail tendon. The specimens were raster scanned in 100 µm steps, in one-dimension (Figs 1 and 2) or two-dimensions (Fig. 3). The X-ray beam can cause localised radiation damage to the tissue, so the scans were performed in such a way that no area of a specimen that had already been irradiated was studied again. All image processing was conducted using Fit2D and Fiberfix software packages.

### X-Ray Talbot interferometry

The X-ray Talbot grating interferometer constructed at the bending magnet beamline BL20B2 at SPring-8 (Momose et al., 2003, 2005), was used to measure the refractive index gradients in whole squid lenses. The monochromatic X-ray beam was tuned to 25 keV with a photon flux energy of 15 keV, which passes through a Si (111) double crystal monochromator, a phase grating and an absorption grating. Phase retrieval was achieved using a five-step fringe-scan method that allowed 10 projections to be captured in 177 s; 600 projections were made per lens. Squid lenses were set in a 2% agarose gel dissolved in a physiologically balanced buffer (Momose et al., 2003) within a specially constructed cell that was placed in the synchrotron beam. Phase shifts were calibrated against solutions of known density and values compared to experimentally derived phase shifts values per pixel in order to determine refractive index (Momose et al., 2003). X-ray Talbot interferometry has been shown to be reproducible with no damage caused to the lens (Pierscionek et al., 2015).

### Transmission electron microscopy (TEM)

Two isolated squid lenses were photographed (Fig. 1S) and immersed in 2.5% glutaraldehyde/2% paraformaldehyde fixative, pH 7.2, overnight after which they were transferred to buffer for five days. Each lens was then removed from buffer, bisected sagittally and the resultant hemispheres returned to fresh fixative for a further 12 h, after which they were washed in several buffer rinses to remove excess fixative and postfixed in 1% osmium tetroxide for 1.5 h. Specimens were then washed in distilled water and contrasted for 1 h in 0.5% aqueous uranyl acetate. They were then dehydrated via a graded series of dilutions to 100% ethanol and embedded in Araldite CY212 resin. Ultrathin sections from the anterior segment were cut in a single plane parallel to the direction of the lens fibres. These were contrasted with uranyl acetate and lead citrate and examined in a Jeol JEM 1010 transmission electron microscope. All measurements were made using ImageJ software.

### Computer modelling

The modelling of the diffraction patterns was guided by our electron micrographs (e.g. Fig. 5B and C). The most striking features are pairs of electron dense transversal bands running parallel to each other about 20 nm apart. Density profile measurements along a direction perpendicular to the bands showed that the profile of each band closely resembled that of a Gaussian function. Although the separation between the bands within a pair was very regular, there seemed to be little regularity in the distances between pairs. We therefore created a parametrised model of these features consisting of two adjacent identical Gaussian functions with their standard deviation (σ) and the distance between their maxima being the only two parameters. The profiles thus obtained (representing one-dimensional electron densities of the bands) were then Fourier transformed to obtain a one-dimensional calculated diffraction pattern.

The experimental diffraction pattern used to optimise the model was obtained by summing together the one-dimensional density profiles through the centre and the maxima of 11 diffraction patterns taken a few millimetres apart from scan A. A background for this pattern was obtained by summing together one-dimensional density profiles taken through the centre but away from the maxima of each pattern. The one-dimensional calculated diffraction patterns were compared to the background-subtracted experimental profiles. This was done for all values of σ in the model ranging between 0 and 20 nm, in steps of 0.01 nm, and for all values of the distance between maxima ranging between 0 and 50 nm, in steps of 0.01 nm. The values for σ and distance that gave the best fit between calculated and experimental one-dimensional patterns were 1.97 and 19.43 nm, respectively (Fig. 6).

## Supplementary Material

10.1242/biolopen.060445_sup1 Supplementary information
