# Supplementary material for: Membrane structures and functional correlates in the bi-segmented eye lens of the cephalopod
Source: Biol Open. 2024 Aug 29;13(9):bio060445. doi: 10.1242/bio.060445 (PMC11381927; doi:10.1242/bio.060445)
Supplement: Supplementary information [file biolopen-13-060445-s1.pdf]

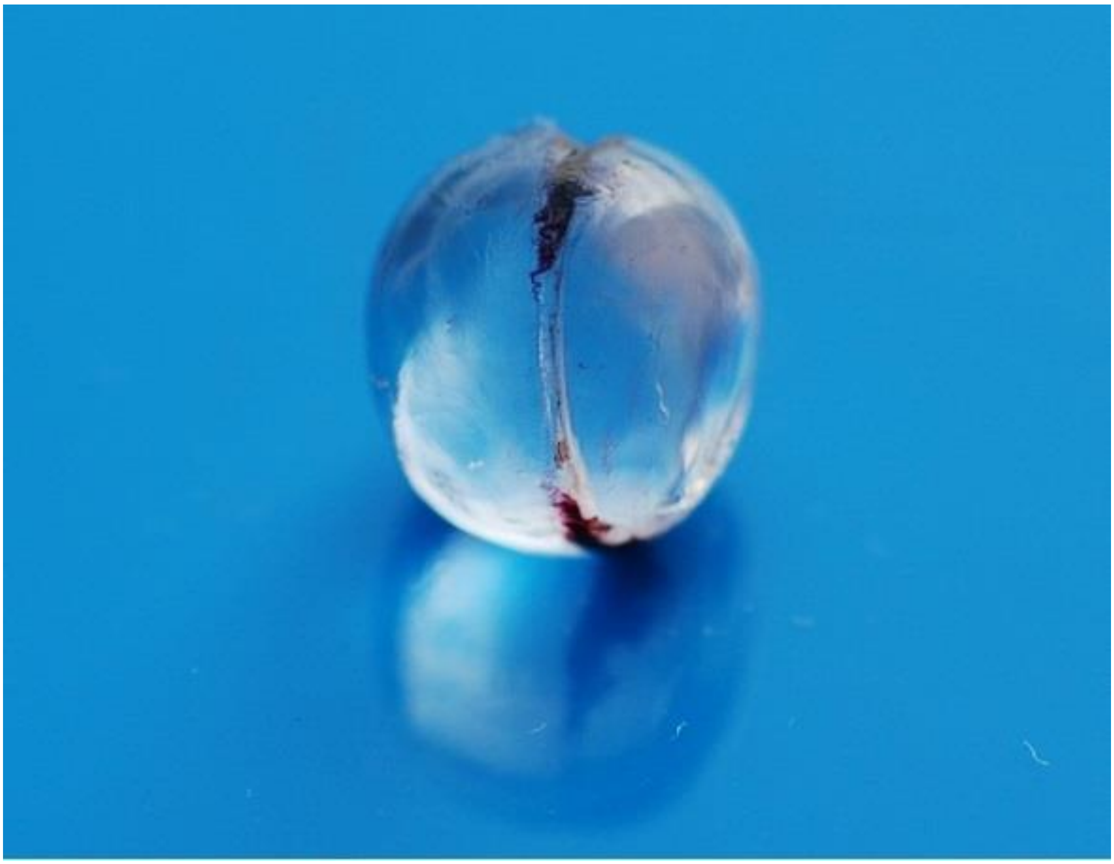

Fig. S1. An image of isolated from the eye of the Japanese flying squid (*Todarodes pacificus*). The anterior plano-convex hemi-spherical segment is on the right of the image, the larger posterior sub-spherical segment to the left.

Biology Open • Supplementary information

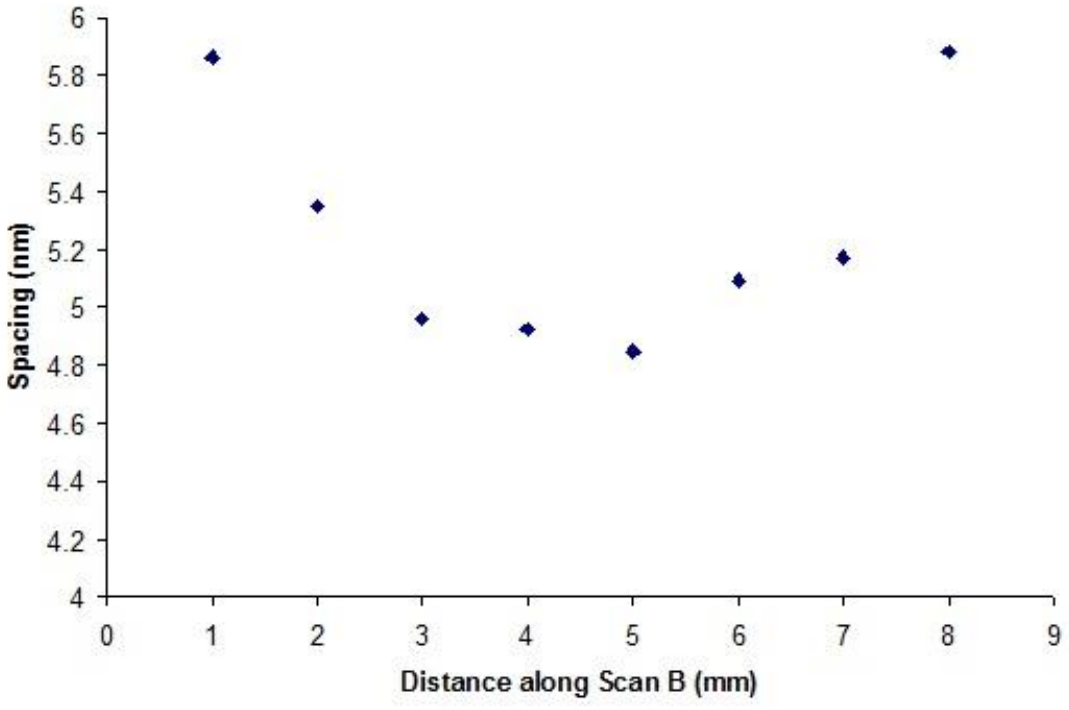

Fig. S2. The spacings of the interference function taken from the summary images of the one-dimensional Scan B as a function of distance along the length of the scan.

Biology Open • Supplementary information

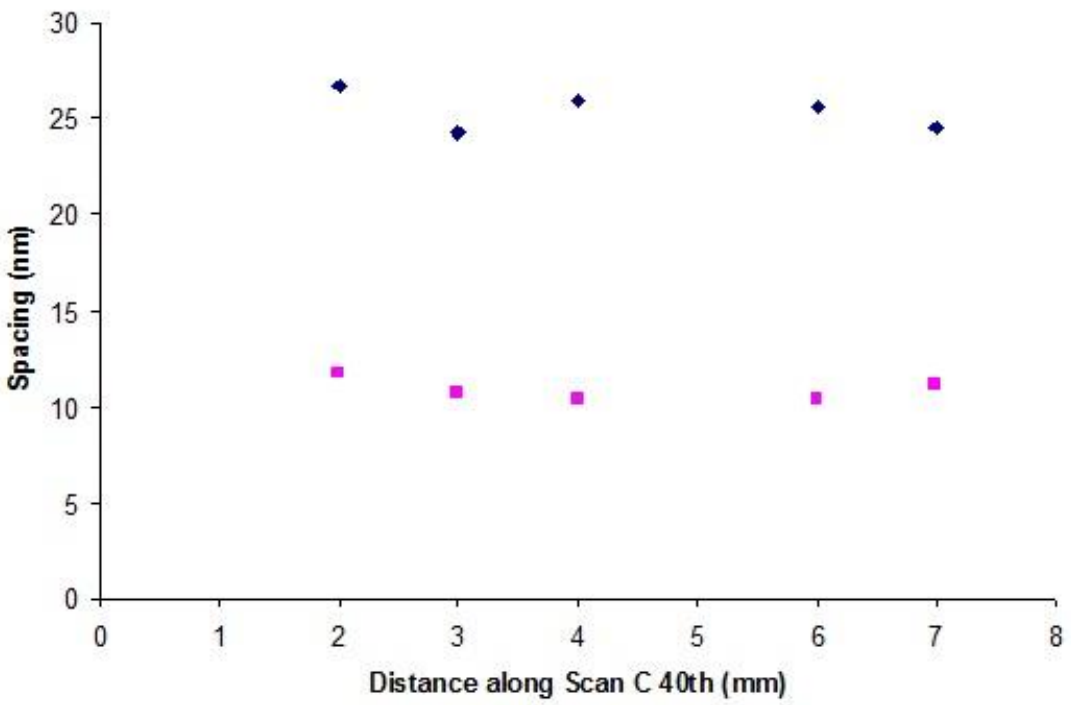

Fig. S3. The spacings of the second (blue diamonds) and third (pink squares) anisotropic reflections, measured from the summary images of the vertical scan in the 3 mm position on the *x* axis (Scan C) plotted as a function of distance along the length of the scan.

Biology Open • Supplementary information

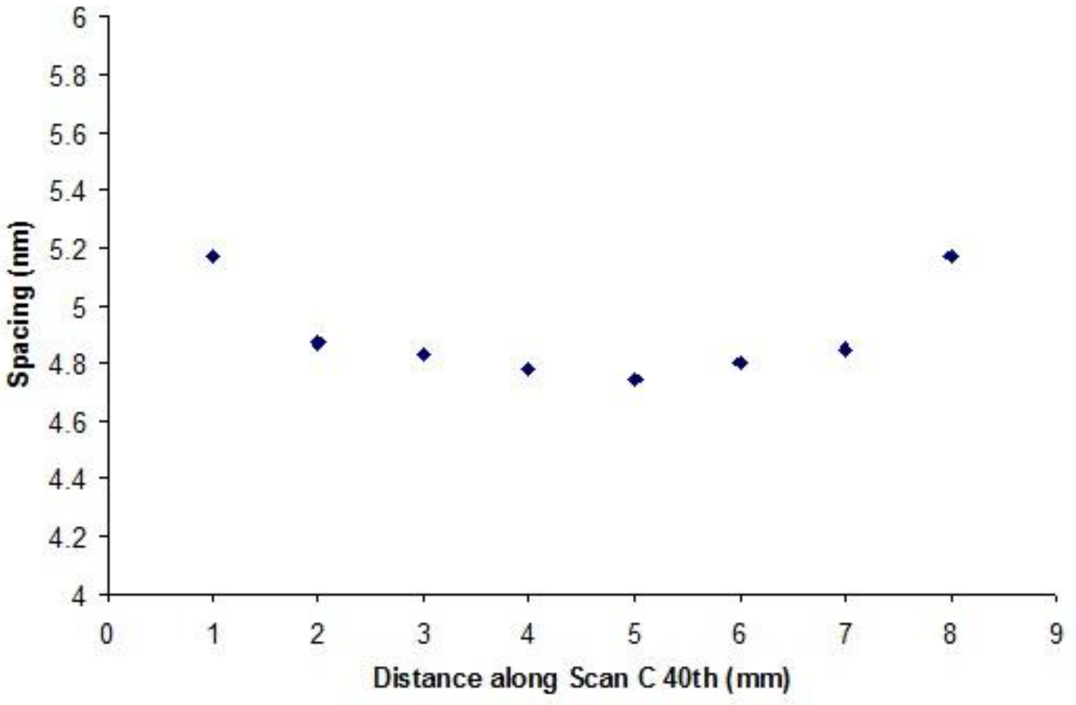

Fig. S4. The spacing of the interference function, measured from the summary images of the 3 mm position on the *x* axis (Scan C) plotted as a function of distance along the length of the scan

Biology Open • Supplementary information

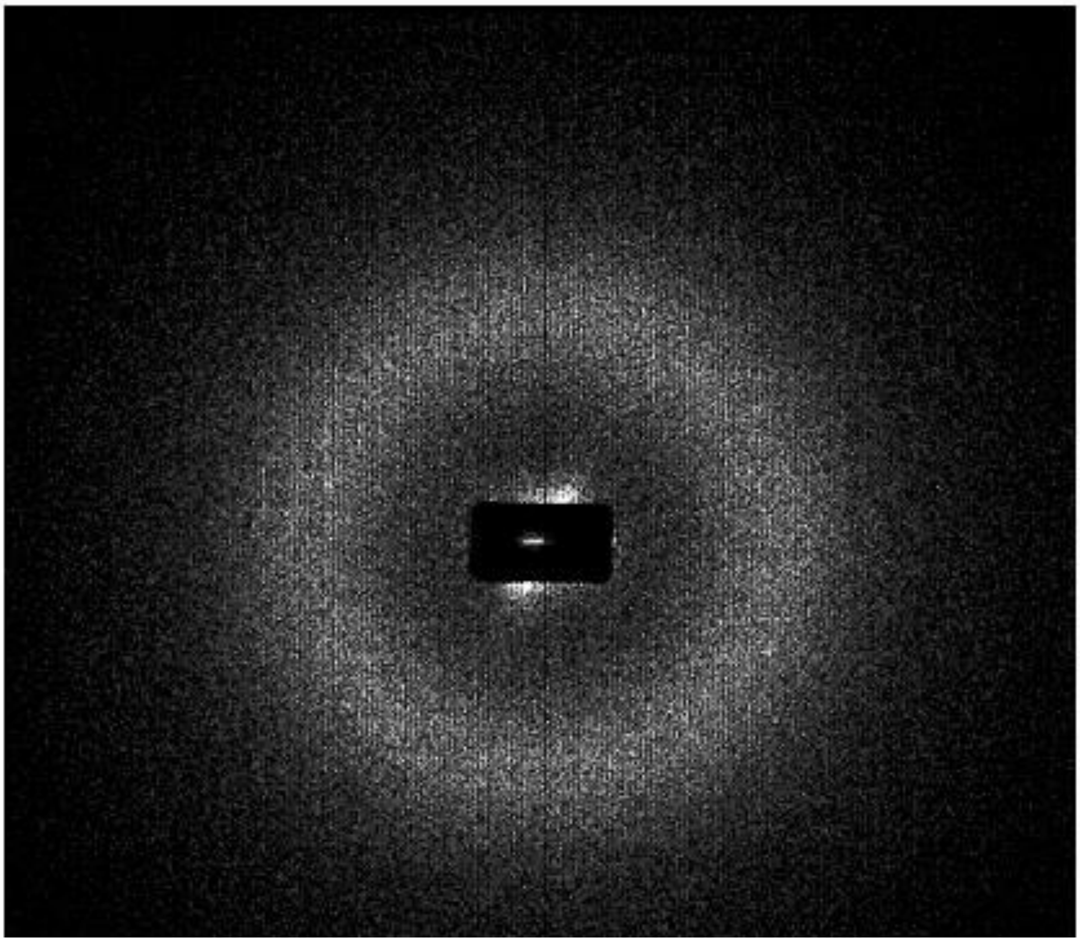

Fig. S5. A low-angle X-ray diffraction pattern from a fresh bovine lens showing the diffuse interference function ring.

Biology Open • Supplementary information

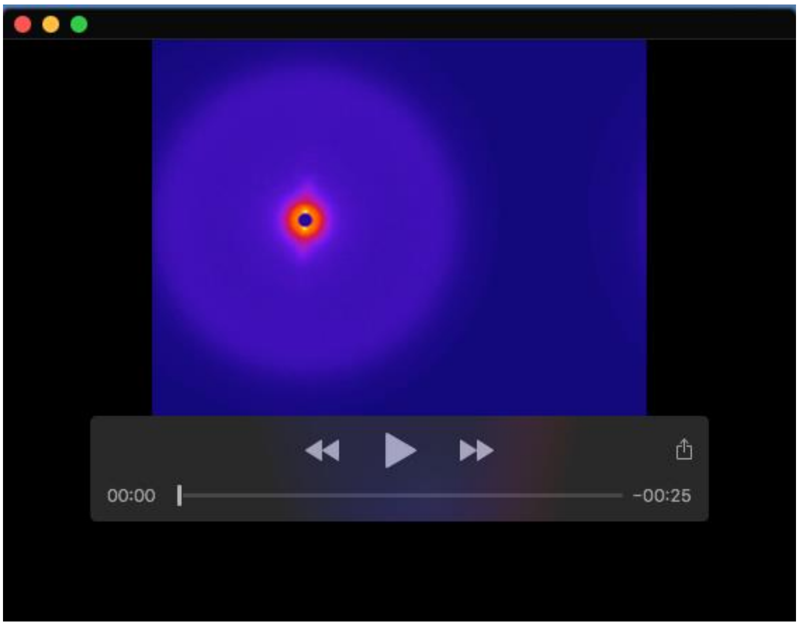

Movie 1. Scan A (one-dimensional), All the X-ray diffraction patterns from the whole squid lens with the beam orthogonal to the optical axis passing through the anterior and posterior segments consecutively, down the meridian of the lens.

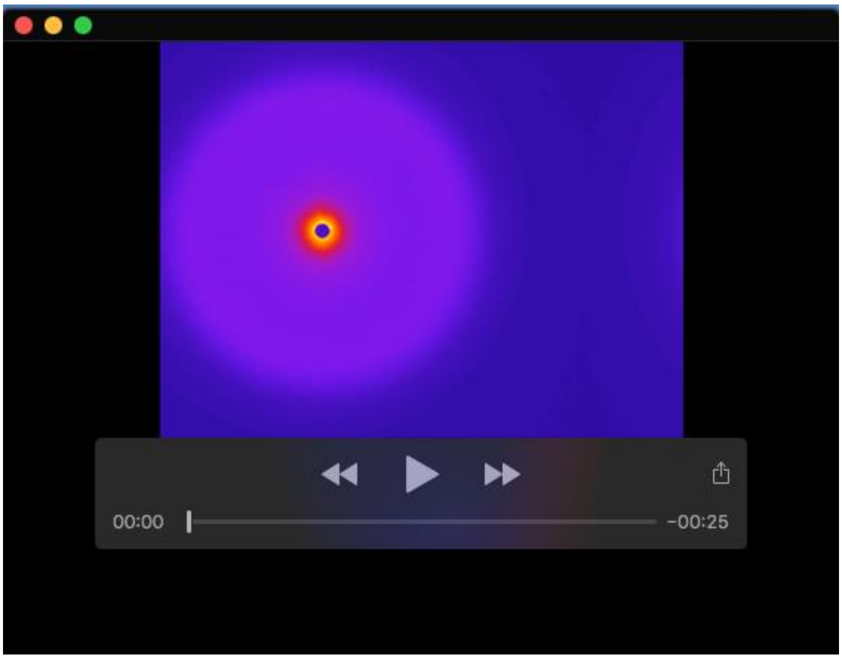

Movie 2. Scan B (one-dimensional), All the X-ray diffraction patterns taken along the meridian of the isolated anterior segment of the squid lens with the X-ray beam parallel with the optical axis.

Biology Open • Supplementary information

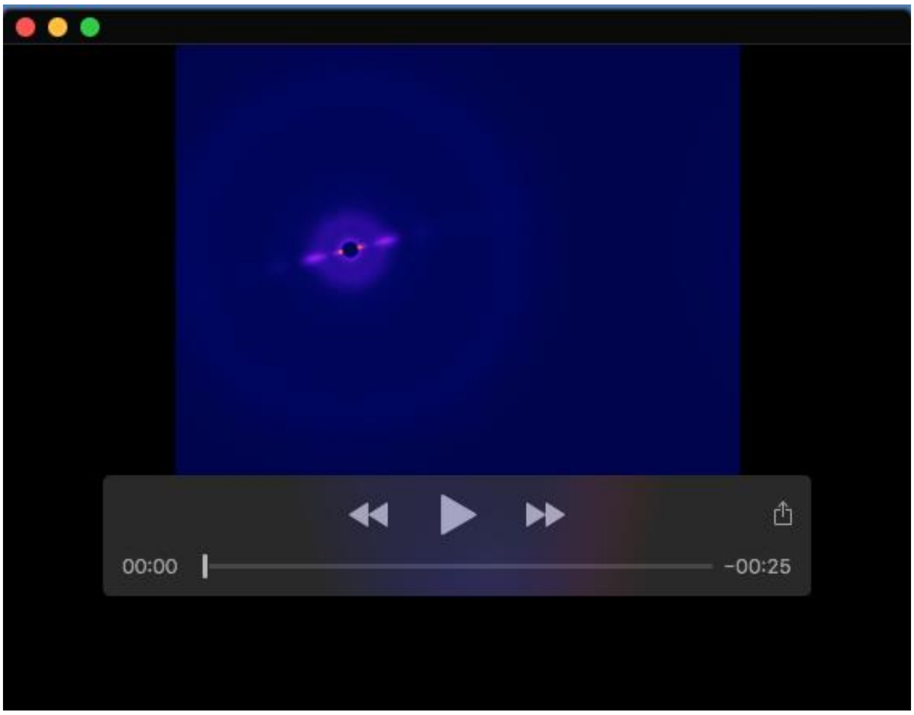

Movie 3. Scan C, All the X-ray diffraction patterns of scan at the 1, 2, 3, 4, and 5 mm positions along the x axis in the y direction of the two-dimensional scan of the isolated posterior segment of the squid lens, with the X-ray beam parallel to the optical axis of the lens.
